# Retraction: Mir-124 attenuates STAT3-mediated TH17 differentiation in colitis-driven colon cancer

**DOI:** 10.3389/fonc.2024.1506202

**Published:** 2024-10-17

**Authors:** Frontiers Editorial Office

The journal retracts the 2020 article cited above.

Following publication, concerns were raised regarding the integrity of the images in the published figures. An investigation by our internal Research Integrity department, conducted in accordance with Frontiers policies, was unable to confirm the validity of the data presented and we cannot vouch for its reliability. The article is therefore retracted.

This retraction was approved by the Chief Executive Editor of Frontiers. The authors received a communication regarding the retraction and had a chance to respond. This communication has been recorded by the publisher.

